# Taxonomic diversity of microbial communities in the cold sulfur spring Bezymyanny (Pribaikalsky district, Republic of Buryatia)

**DOI:** 10.18699/vjgb-25-30

**Published:** 2025-04

**Authors:** T.G. Banzaraktsaeva, E.V. Lavrentyeva, V.B. Dambaev, I.D. Ulzetueva, V.V. Khakhinov

**Affiliations:** Institute of General and Experimental Biology of the Siberian Branch of the Russian Academy of Sciences, Ulan-Ude, Russia; Institute of General and Experimental Biology of the Siberian Branch of the Russian Academy of Sciences, Ulan-Ude, Russia D. Banzarov Buryat State University, Ulan-Ude, Russia; Institute of General and Experimental Biology of the Siberian Branch of the Russian Academy of Sciences, Ulan-Ude, Russia; Baikal Institute of Nature Management of the Siberian Branch of the Russian Academy of Sciences, Ulan-Ude, Russia; D. Banzarov Buryat State University, Ulan-Ude, Russia

**Keywords:** cold sulfur spring, microbial community diversity, microbial mat, bottom sediment, water, sulfur-oxidizing bacteria, sulfate-reducing bacteriaFor, холодный сероводородный источник, разнообразие микробных сообществ, микробный мат, донный осадок, вода, сероокисляющие бактерии, сульфатвосстанавливающие бактерии

## Abstract

The environmental conditions of cold sulfur springs favor the growth and development of abundant and diverse microbial communities with many unique sulfur cycle bacteria. In this work, the taxonomic diversity of microbial communities of three different biotopes (microbial mat, bottom sediment, and water) in the cold sulfur spring Bezymyanny located on the shore of Lake Baikal (Pribaikalsky district, Republic of Buryatia) was studied using high-throughput sequencing of the 16S rRNA gene. By sequencing the microbial mat, bottom sediment, and water samples, 76,972 sequences assigned to 1,714 ASVs (ASV, amplicon sequence variant) were obtained. Analysis of the ASV distribution by biotopes revealed a high percentage (66–93 %) of uniqueness in the three communities studied. An estimate of the alpha diversity index showed that bottom sediment community had higher indices, while microbial mat community was characterized by a lowest diversity. Bacteria of the phyla Pseudomonadota, Bacteroidota, Campylobacterota, Actinomycetota, Desulfobacterota dominated in different proportions in the studied communities. The features of the community structure of the studied biotopes were established. The microbial mat community was represented mainly by Thiothrix (43.2 %). The bottom sediment community was based on Sulfurovum (11.2 %) and co-dominated by unclassified taxa (3.2–1 %). Sequences assigned to the genera Novosphingobium, Nocardioides, Legionella, Brevundimonas, Sphingomonas, Bacillus, Mycobacterium, Sphingopyxis, Bradyrhizobium and Thiomicrorhabdus were found only in the water microbial community. Sulfur-oxidizing bacteria (SOB) and sulfate-reducing bacteria (SRB) were identified in all the communities studied, which indicates the ongoing processes of the sulfur cycle in the Bezymyanny spring ecosystem. It should be noted that sequences of unclassified and uncultivated sulfur cycle bacteria were present in all communities and a significant proportion of sequences (20.3–53.9 %) were not classified.

## Abstract (ru)

Экологические условия холодных серосодержащих источников благоприятствуют росту и развитию богатых микробных сообществ со множеством уникальных бактерий цикла серы. В настоящей работе с использованием высокопроизводительного секвенирования гена 16S рРНК было изучено таксономическое разнообразие микробных сообществ трех различных биотопов (микробный мат, донный осадок и вода) в холодном сероводородном источнике Безымянный, расположенном на побережье озера Байкал (Прибайкальский район, Республика Бурятия). В результате секвенирования проб микробного мата, донного осадка и воды получено 76 972 последовательности, отнесенных к 1714 ASV (amplicon sequence variant – варианты последовательностей ампликонов). Анализ распределения ASV по биотопам выявил высокий показатель (66–93 %) уникальности трех исследуемых сообществ. Оценка индекса альфа-разнообразия показала, что сообщество донного осадка имело более высокие индексы, сообщество микробного мата отличалось наименьшим разнообразием. В исследуемых сообществах в разных пропорциях доминировали бактерии филумов Pseudomonadota, Bacteroidota, Campylobacterota, Actinomycetota, Desulfobacterota. Установлены особенности структуры сообществ исследуемых биотопов. Сообщество микробного мата было представлено преимущественно бактериями рода Thiothrix (43.2 %). В сообществе донного осадка основу составляли бактерии рода Sulfurovum (11.2 %), содоминировали неклассифицируемые таксоны (3.2–1 %). Микробное сообщество воды характеризовалось присутствием последовательностей, обнаруженных только в воде. Данные последовательности были отнесены к родам Novosphingobium, Nocardioides, Legionella, Brevundimonas, Sphingomonas, Bacillus, Mycobacterium, Sphingopyxis, Bradyrhizobium и Thiomicrorhabdus. Во всех изучаемых сообществах были идентифицированы сероокисляющие бактерии (SOB) и серовосстанавливающие бактерии (SRB), что свидетельствует о протекающих процессах цикла серы в экосистеме источника Безымянный. Необходимо отметить, что во всех сообществах присутствовали последовательности неклассифицированных и некультивируемых бактерий цикла серы, и в целом значительную долю последовательностей (20.3–53.9 %) не удалось классифицировать

## Introduction

The Baikal region has a large area of natural water bodies, among which a significant part is represented by numerous vents of mineral springs. On the territory of Buryatia there are almost all known types of mineral waters, which are formed in the hypergenesis zone of rock (Borisenko, Zamana, 1978; Namsaraev et al., 2005). Sulfur springs are enriched with hydrogen sulfide as a result of biochemical sulfate reduction coming with mineral water or mineral suspension containing sulfates in exchange and bound state (Borisenko, Zamana, 1978; Kononov, 1983).

The environmental conditions of cold sulfur springs favor the growth and development of abundant and diverse microbial communities with many unique sulfur cycle bacteria (Douglas S., Douglas D., 2001; Rudolph et al., 2004; Chaudhary et al., 2009; Headd, Engel, 2014; Hahn et al., 2022). Cold springs are characterized by a slow change of parameters such as pH, temperature, dissolved gases and other factors and are more stable for bacterial life compared to other environments (Nosalova et al., 2023c).

Previous studies in cold sulfur springs, using culturable and non-culturable approaches, have mainly focused on the composition and community structure of microbial mats (Douglas S., Douglas D., 2001; Chaudhary et al., 2009; Klatt et al., 2016; Sapers et al., 2017; Nosalova et al., 2023b). Microscopy study in microbial mats from the cold sulfur spring Ancaster (Ontario, Canada) showed the development of all major groups of sulfide-oxidizing bacteria, purple, green, cyanobacteria and colorless sulfur-oxidizing bacteria (Douglas D., Douglas S., 2001). Using 16S rRNA gene sequencing, a clear dominance of phyla Pseudomonadota and Campylobacterota was shown in microbial mats of Slovak cold sulfur springs, and genera Thiothrix and Sulfurovum were identified as the core microbiota of microbial mats (Nosalova et al., 2023b).

In the present work, the taxonomic diversity of microbial communities of microbial mat, bottom sediment, and water in the cold sulfur spring Bezymyanny, located on the coast of Lake Baikal, was studied for the first time using high-throughput sequencing of the 16S rRNA gene. The aim of the study was to determine the bacterial composition of microbial communities in different biotopes of the Bezymyanny spring (Pribaikalsky district, Republic of Buryatia).

## Materials and methods

**Fig. 1.**
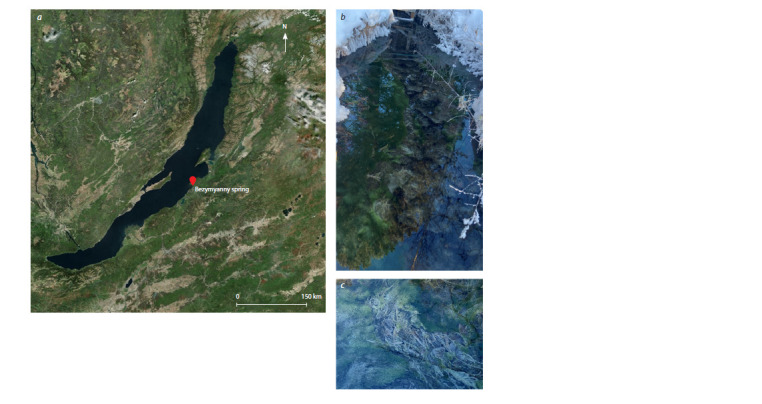
Map-scheme of Bezymyanny spring location (a), Bezymyanny spring photo (b), spring microbial mat photo (c).

The Bezymyanny sulfur spring is located in the forest area close to the coastline of Lake Baikal and is situated at an altitude of 638 m above sea level (53°02ʹ48.95ʺ N, 108°19ʹ57.68ʺ E) (Fig. 1). The water of the mineral spring seeps through a 25–30 cm thick layer of loose sediments to form a stream. In the stream, microbial mats were found on the surface of the bottom sediments.

Microbial mat, bottom sediment and water samples were collected in October, 2023. Water samples for chemical analysis were collected from the mineral spring vent and the outflow stream, into clean polyethylene and sterile glass bottles. Microbial mat, bottom sediment and water samples for microbiological studies were collected into sterile 50 ml Falcon tubes

At the sampling sites, pH and temperature were measured with a portable pH-meter with a sensor thermometer pH-200 HM Digital (South Korea). Total salinity was measured with a COM-100 conductometer. A portable redox potential meter ORP (Portugal) was used to determine redox potential. The concentration of carbonates and hydrocarbonates in the analyzed waters was determined in the field environment at the moment of sampling by titration with 0.1 N HCl in the presence of phenolphthalein and methyl orange indicators. Total hardness, calcium and magnesium ions, chloride ions were determined by titrimetric method. Concentrations of ammonium ions, nitrite, nitrate, phosphate, sulfate and fluoride ions, silicic acid were determined by spectrophotometric method. A single beam spectrophotometer CECIL 1000 (UK) was used for analysis; ion concentrations were calculated by regression equations. The content of hydrogen sulfide and sulfides was determined by calorimetric method with the addition of iron-ammonium alum at 670 nm. Sulfites and thiosulfates were determined by reverse iodometric titration (Fomin, 2000).

In accordance with the manufacturer’s instructions, a reagent kit (MACHEREY-NAGEL NucleoSpin Soil) from MACHEREY-NAGEL (Duren, Germany) was used to isolate DNA from microbial mat, sediment and water samples.

Purified DNA preparations were used to create libraries of 16S rRNA gene fragments by PCR using universal primers for V4 variable region: F515/R806 (GTGCCAGCMGCCG GCGGTAA/GGACTACVSGGGTATCTAAT) (Bates et al., 2011), with attached adapters and unique Illumina barcodes. PCR was carried out in 15 μL of reaction mixture containing 0.5–1 unit of activity of Q5 High-Fidelity DNA Polymerase (NEB, Ipswich, MA, USA), 5 pM each of forward and reverse primers, 10 ng of DNA matrix and 2 nM of each dNTP (LifeTechnologies, Carlsbad, CA, USA). The mixture was denatured at 94 °C for 1 min, followed by 35 cycles of 94 °C for 30 s, 50 °C for 30 s and 72 °C for 30 s. The final elongation was carried out at 72 °C for 3 min. PCR products were purified according to the method recommended by Illumina using AMPureXP magnetic particles (Beckman- Coulter, Brea, CA, USA).

Library preparation and sequencing were carried out in accordance with the manufacturer’s recommendations for operation of the Illumina MiSeq instrument (Illumina, San Diego, CA, USA) using MiSeq® ReagentKit v3 (600 cycles). Initial data processing, namely, sample demultiplexing and removal of adapters, was carried out using Illumina software (Illumina, USA). For subsequent denoising, sequence merging, deletion of chimeric reads, restoration of the original phylotypes (ASV, amplicon sequence variant), and further taxonomic classification of the obtained ASVs, the software packages DADA2 (Callahan et al., 2016), PHYLOSEQ (McMurdie, Holmes, 2013) and SILVA (Quast et al., 2013) were used; the work was carried out in the R software environment. Raw sequences were pre-filtered for quality with the filterAndTrim function with a sequence trim length of 250 and 200 n for forward and reverse reads respectively; the acceptable level of expected error was 2 and 3 for forward and reverse reads respectively. The learnErrors function with MAX_CONSIST parameter equal to 20 was used to build the error model. The dereplication process was carried out using the derepFastq function with parameter n equal to 10e7. Denoising was carried out with the dada function with the pool parameter set to ʻpseudoʼ. The recovered sequences were combined using the mergePairs function. The table of numbers of the obtained phylotypes was constructed using the makeSequenceTable function. A check for the presence of chimeras was conducted and they were filtered using the removeBimeraDenovo function by the ʻconsensusʼ method with the parameter minFoldParentOverAbundance equal to 2 and allowOneOff set to ʻTRUEʼ. The OTU table was constructed using the otu_table function with the taxa_are_ rows parameter set to ʻFALSEʼ. The file with representations for each phylotype was generated using the getSequences, DNAStringSet and writeXStringSet functions

Classification of the resulting phylotypes was performed with the assignTaxonomy function using the SILVA release 128 database and with the minBoot parameter set to 70. Fragments related to plastid and mitochondrial DNA were removed from the list of phylotypes. BIOM table construction was performed using the python 3 programming language, with the biom, numpy and pandas packages. The tools of the QIIME 1 software package (Caporaso et al., 2010) were used to present the data of the taxonomic analysis. The research was carried out at the Core Centrum ‘Genomic Technologies, Proteomics and Cell Biology’ in All-Russia Research Institute for Agricultural Microbiology (St. Petersburg, Russia).

The NCBI server (https://blast.ncbi.nlm.nih.gov/Blast. cgi) was used to search for closest homologues. Alpha diversity indices were calculated using the software package Past 4.16 (Hammer et al., 2001). The iNEXT Online resource (https://chao.shinyapps.io/iNEXTOnline/) was used to plot rarefaction curves (Chao et al., 2014, 2016). The Venn diagram and heat map were created with the SRplot Online resource using the pheatmap R package, where a standard scaling approach (https://www.bioinformatics.com.cn/ plot_basic_cluster_heatmap_plot_024_en) was applied to normalize the data (Tang et al., 2023).

## Results

Physical and chemical characteristics of the Bezymyanny spring

At the time of sampling, the water temperature was 5.7 °C, the pH value, 8.4, mineralization, 0.12 g/dm3 and redox potential, –113 mV. Hydrochemical analysis of water composition showed hydrogen carbonate and chloride ions content of 30.5 and 3.5 mg/dm3 respectively. Calcium cations were 36.6 mg/dm3 and magnesium cations were 15.6 mg/dm3. Nitrate and nitrite ions in the amount of 4.7 and 0.01 mg/dm3 and phosphate and fluoride ions in the amount of 0.35 and 0.32 mg/dm3 were found. Carbonate and ammonium ions were not detected and iron ions were present in the amount of 0.14 mg/dm3. Of the sulfur-containing compounds, 42.0 mg/dm3 of sulfate ions, 11.0 mg/dm3 of sulfide ions, and the presence of small amounts of sulfite and thiosulfate ions were detected.

Analysis of microbial communities diversity in different biotopes

**Fig. 2.**
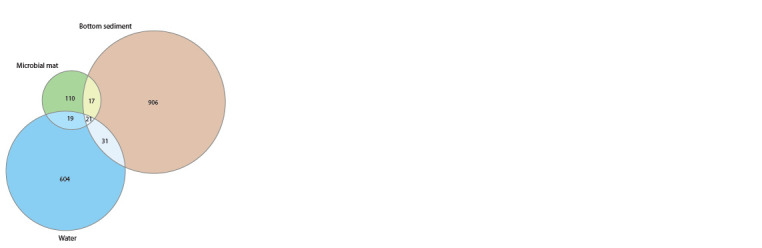
Venn diagram of ASV bacterial communities of microbial mat, bottom sediment and water of the Bezymyanny spring.

Pyrosequencing of the 16S rRNA gene fragment from microbial mat, bottom sediment and water samples produced a total of 143,192 sequence reads. After their filtration, alignment, pre-clustering and removal of chimeric sequences and singletons, 76,972 reads were included in the analysis. The sequences were assigned to 1,714 ASVs and their distribution in three biotopes of the Bezymyanny spring is shown in the Venn diagram (Fig. 2). Only 21 ASVs were shared for all three biotopes. Most of the microbial communities in the biotopes are represented by unique sequences

Alpha diversity indices were evaluated to assess diversity in each sample (Table 1). The bottom sediment microbial community was characterized by the highest number of ASVs, the highest diversity, and its unique ASVs constituted 93 %.

**Table 1.**
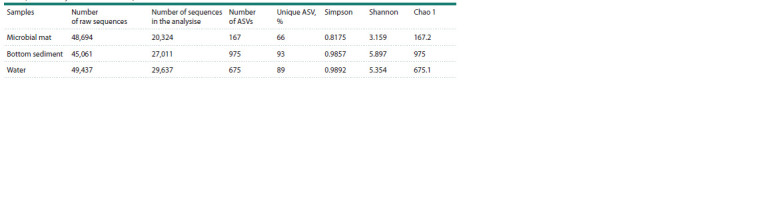
Number of sequences and ASVs in the Bezymyanny spring samples and alpha diversity indices of the samples

**Fig. 3.**
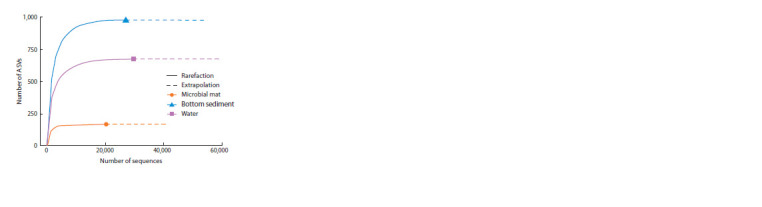
ASV rarefaction curves in the samples of microbial mat, bottom sediment and water

The rarefaction curves showed similar results as in Table 1 (Fig. 3).

Taxonomic composition of the microbial community

**Fig. 4.**
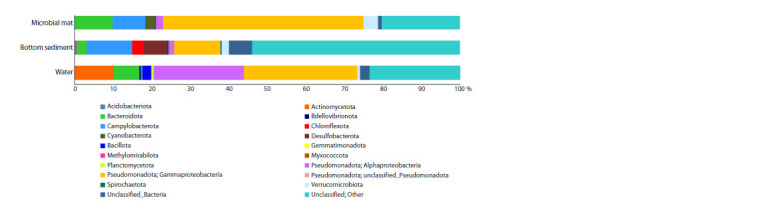
Taxonomic diversity of prokaryotes at the phylum level (classes for Pseudomonadota) in microbial mat, bottom sediment and water of Bezymyanny spring

Microbial communities of microbial mat, bottom sediment and water of the Bezymyanny sulfide spring are represented by the Bacteria domain. Single sequences were assigned to the Archaea domain. Representatives of the phylum Pseudomonadota dominated in all samples (12.9–53.8 % of the total number of reads) (Fig. 4).

Pseudomonadota were represented by Gammaproteobacteria, which formed the core of the microbial communities (52 % in microbial mat, 29.4 % in water and 11.9 % in bottom sediment). The number of Alphaproteobacteria ranged from 0.9 % in bottom sediment to 23.4 % in water. Bacteroidota (2.3–9.8 %) was found in all samples. Sequences of the phylum Campylobacterota were widely distributed in the microbial mat and bottom sediment, 8.5 and 11.8 %, respectively. The water community was characterized by the high abundance of Actinomycetota (10.1 %) and Bacillota (2.2 %). The mat community was characterized by a high proportion of Verrucomicrobiota (3.8 %) and Cyanobacterota (2.6 %). The bottom sediment community showed a high percentage of Desulfobacterota (6.3 %) and Chloroflexota (2.9 %). It should also be noted that in all communities, 0.9–6 % of sequences were identified to the domain level, and a significant proportion of sequences (20.3–53.9 %) were not classified.

**Fig. 5.**
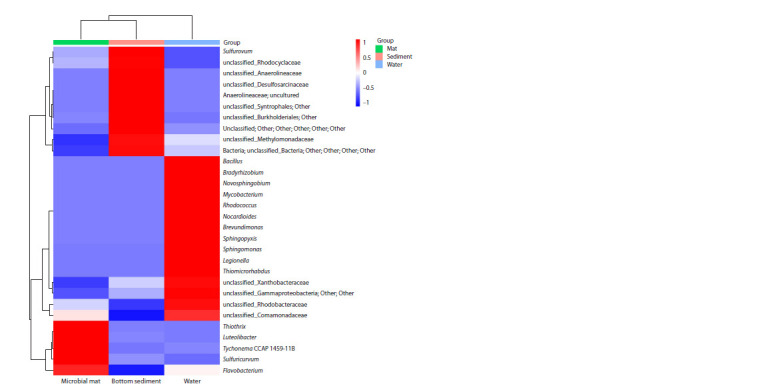
Heat map of taxa (number of reads ≥ 1 %) in microbial mat, bottom sediment and water of the Bezymyanny spring. The color scale reflects the distance of each value from the mean in the standard deviation units

The analysis of taxonomic composition at a deeper level showed that representatives of the genera Sulfuricurvum, Sulfurovum, Thiothrix, Flavobacterium and unclassified sequences of unclassified_Comamonadaceae, unclassified_ Burkholderiales, unclassified_Gammaproteobacteria were present in all investigated microbial communities. However, representatives of these taxa were un-evenly distributed over communities and varied from clear dominance to representation of 0.1 % or less. It was found that each microbial community had its own characteristics at the level considered (Fig. 5).

The microbial mat community was heavily dominated by bacteria of the genus Thiothrix (43.2 %). The mat community also included bacteria of genus Flavobacterium, Sulfuricurvum, Luteolibacter and cyanobacteria Tychonema CCAP 1459-11B. The core bottom sediment community was formed by Sulfurovum (11.2 %) and representatives of unclassified_ Burkholderiales, unclassified_Anaerolineaceae, unclassified_Desulfosarcinaceae, unclassified_Rhodocyclaceae, unclassified_Methylomonadaceae, unclassified_Syntrophales co-dominated (3.2–1 %). In the water community, among the dominants (>1 % of all sequences obtained), sequences typical only for this community and assigned to the genera Novosphingobium, Nocardioides, Legionella, Brevundimonas, Sphingomonas, Bacillus, Mycobacterium, Sphingopyxis, Bradyrhizobium and Thiomicrorhabdus were present.

Sulfur cycle bacteria

Microorganisms involved in the sulfur cycle were found in the taxonomic composition of the Bezymyanny spring. Thiothrix and Sulfurovum comprised the majority of the sequences in all of the investigated spring biotopes. Representatives of the genus Thiothrix of the family Thiotrichaceae, class Gammaproteobacteria, were the key taxon in the microbial mat (43.2 %). Genus Sulfurovum of the family Sulfurovaceae, class Campylobacteria, constituted a significant part (11.2 %) of the taxonomic composition of the bottom sediment community (Table 2).

**Table 2.**
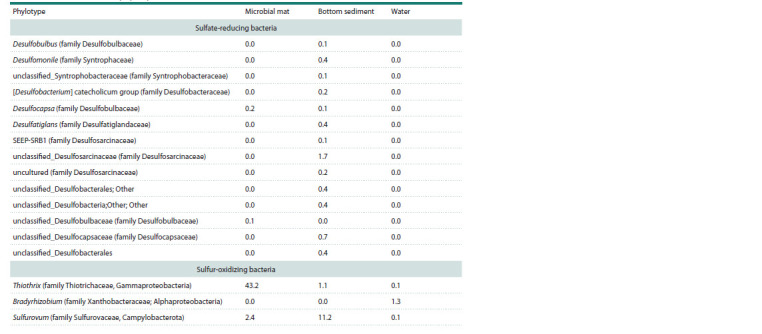
Comparative characteristics of sulfur cycle bacteria representation in microbial communities of Bezymyanny spring biotopes (number of sequences, %)

The identified sulfate-reducing bacteria belong mainly to uncultivated unclassified_Desulfosarcinaceae (family Desulfosarcinaceae) and reach 1.7 % in bottom sediments. Other representatives of sulfate-reducing bacteria account for <1 % in the bottom sediment and microbial mat and are assigned to the genera Desulfobulbus, Desulfomonile, Desulfocapsa, Desulfatiglans (family Desulfatiglandaceae) and unclassified_Syntrophobacteraceae, [Desulfobacterium] catecholicum group (family Desulfobacteraceae).

## Discussion

The territory of the Republic of Buryatia is extremely rich in mineral waters with different physical properties, chemical and gas composition (Mikhailov, Tolstikhin, 1946; Tkachuk et al., 1957; Namsaraev et al., 2005). Cold mineral springs are formed both as a result of interaction of water with the host rocks, and owing to input of some constituents from deep zones of the earth. The Bezymyanny sulfur spring is characterized by regular low temperature with reduced conditions. Water is alkalescent and weakly mineralized with predominance of hydrocarbonate, sulfate and sulfide ions.

For the first time, studies of microbial mat, bottom sediment and water by 16S rRNA gene sequencing in the cold sulfur spring Bezymyanny revealed 15 bacterial phyla, five of which were most abundant: Pseudomonadota, Bacteroidota, Campylobacterota, Actinomycetota, Desulfobacterota. Pseudomonadota, represented by class Gammaproteobacteria, and Bacteroidota were dominant in all samples, which coincides with previous reports on the communities of different types of sulfur habitats (Elshahed et al., 2003; Gulecal-Pektas, Temel, 2016; Nosalova et al., 2023a). The chemolithotrophic Gammaproteobacteria and Bacteroidota play a major role in the formation of primary production by iron and sulfide oxidation in inactive sulfide ecosystems (Dong et al., 2021). Campylobacterota were present in all samples and dominated in the bottom sediment (11.8 %) and microbial mat (8.5 %) communities. Similar results were obtained when communities in hydrothermal vents and aphotic (cave) sulfide springs were studied, where it was shown that in these ecosystems, sulfur-based chemolithoautotrophy is mainly carried out by Campylobacterota (formerly Epsilonproteobacteria) (Karl et al., 1980; Sarbu et al., 1996; Engel et al., 2003, 2004).

A distinctive feature of the Bezymyanny spring water community was the significant presence of Actinomycetota representatives. Some new actinobacteria from geothermal environments are known to be able to grow autotrophically with sulfur as an energy source (Norris et al., 2011). Using a culturable approach, sulfur-oxidizing bacteria phylogenetically related to Actinomycetota have been isolated from cold, high sulfide and sulfate springs at Gypsum Hill (Arctic, Canada) (Perreault et al., 2008). In the bottom sediment of the Bezymyanny spring, Desulfobacterota (6.3 %) made a significant contribution to the community. The abundance of sulfate-reducing bacteria belonging to Desulfobacterota has been described in a number of publications on the microbiota of cold saline springs in the Canadian Arctic and has also been found in the high-sulfide wetland Solodovka (Samara region, Russia) (Perreault et al., 2008; Sapers et al., 2017; Colangelo-Lillis et al., 2019; Gorbunov et al., 2022).

It has now been noted that cold sulfur springs harbor unique, not yet explored bacterial communities (Hamilton et al., 2015; Nosalova et al., 2023a). A high number of sequences in our studies remained unclassified, suggesting the presence of many undiscovered and unstudied communities and indicating potentially novel microorganisms in the cold sulfur spring ecosystem.

Analyses of the taxonomic composition of microbial communities at the genus level showed characteristic features of each community in all three biotopes studied. The microbial mat community was represented mainly by bacteria of the genus Thiothrix (43.2 %). NCBI database sequence analysis revealed similarity (100 % homology) with Thiothrix fructosivorans, which is able to deposit intracellular elemental sulfur in the presence of reduced inorganic sulfur compound (Howarth et al., 1999). A recent study on the Baikal region described an unculturable Thiothrix sp. from the mixing zone of the waters of Lake Baikal and the Zmeiny geothermal spring (northern basin of Lake Baikal, Russia) (Chernitsyna et al., 2024). Comparative analysis of Thiothrix sequences from our study and Thiothrix from the geothermal spring Zmeiny revealed 99 % similarity. The mat community of the Bezymyanny spring included sequences assigned to the genera Flavobacterium, Sulfuricurvum, Luteolibacter and the cyanobacterium Tychonema CCAP 1459-11B, the closest homologues of which have been isolated mainly from low-temperature habitats (Kodama, Watanabe, 2004; Jiang et al., 2012; Yang et al., 2019; Conklin et al., 2020).

The bottom sediment community was based on bacteria of the genus Sulfurovum (11.2 %), the closest homologue (98.81 %) of which was the mesophilic, facultatively anaerobic sulfur- and thiosulfate-oxidizing Sulfurovum lithotrophicum (Inagaki et al., 2004). Co-dominants with the proportion ranging from 1 to 3.2 % of all sequences were classified only up to the order and family levels. A search for closely related species in the NCBI database for unclassified_Burkholderiales (3.2 % presence) showed 98 % similarity to Georgfuchsia toluolica (Pseudomonadota; Betaproteobacteria; Nitrosomonadales; Sterolibacteriaceae), which is able to use Fe(III), Mn(IV) and nitrate as terminal electron acceptors for growth on aromatic compounds (Weelink et al., 2009). Unclassified_Anaerolineaceae typical for bottom sediments had 89 % homology with the marine thermophilic, anaerobic and heterotrophic bacterium Thermomarinilinea lacunofontalis (Nunoura et al., 2013). The closest homologue (95 % similarity) for unclassified_Desulfosarcinaceae was Desulfosarcina widdelii, hydrocarbon-degrading sulfatereducing bacteria (Watanabe et al., 2017).

In our study, bacteria of the genera Novosphingobium, Nocardioides, Legionella, Brevundimonas, Sphingomonas, Bacillus, Mycobacterium, Sphingopyxis, Bradyrhizobium and Thiomicrorhabdus dominated among the sequences found only in the water community. Representatives of these genera are found in various natural environments and belong to heterotrophic prokaryotes that utilize various carbon, nitrogen and sulfur compounds as energy sources (Fliermans, 1996; Kumar R. et al., 2017; Tóth et al., 2017; Song et al., 2022; Kuang et al., 2023). Bacteria of the genera Novosphingobium, Nocardioides, Sphingomonas and Sphingopyxis are known to be able to grow under low nutrient conditions and are important agents in the biodegradation of various persistent and toxic organic substances, including aromatic compounds, hydrocarbons, halogenoalkanes, nitrogen heterocycles and polymeric polyesters (Song et al., 2022; Ma et al., 2023). In the water community, the closest homologues of the dominant sequences were bacteria involved in the sulfur cycle. For example, sequences assigned to the genus Bacillus showed 100 % similarity to the chemolithoautotrophic thiosulfate-oxidizing bacterium Bacillus thioparus (Pérez-Ibarra et al., 2007). Sequences identified as Thiomicrorhabdus found close affinity to Thiomicrorhabdus aquaedulcis, freshwater obligate sulfuroxidizing chemolithotroph (Kojima, Fukui, 2019).

Microbial oxidation and reduction of sulfur are the most active and ancient metabolic processes in the sulfur cycle that occur in various ecosystems. These processes are carried out by sulfur-oxidizing (SOB) and sulfate-reducing bacteria (SRB) in all ecosystems and are considered as a key phenomenon in the biogeochemical sulfur cycle (Kumar U. et al., 2018). At the genus level, the microbial mat of the Bezymyanny sulfur spring was found to be predominantly composed of the colorless sulfur-oxidizing bacteria Thiothrix. Thiothrix species are considered to be typical of sulfur-oxidizing microbial communities in sulfur-rich habitats. Using a non-cultivation approach, the genus Thiothrix was identified in cold sulfur springs in Slovakia (Nosalova et al., 2023b).

The bottom sediment was dominated by members of the genus Sulfurovum. Previous studies have found this genus to be part of the microbial community in a variety of sulfur environments including springs, caves, vents and oxygenfree sediments (Nosalova et al., 2023b). These facultative anaerobic chemolithotrophs succeed under extreme conditions and are primary producers in sulfur-rich habitats. In the work of Wright et al. (2013), it was noted that all sulfur redox genes are present in the genomes of sequenced representatives of Sulfurovum and their genetic ability allows them to use various sulfur compounds.

In addition, obligate chemolithoautotrophic sulfur-oxidizing bacterial species related to Thiomicrorhabdus have been found only in cold spring water. Thiomicrorhabdus has previously been found in cold saline environments, including Arctic marine sediments and Antarctic subglacial brines (Knittel et al., 2005). Thiomicrorhabdus has also been found in abundance in cold saline spring sediments on Axel Heiberg Island, Canada (Magnuson et al., 2023). The authors mark that Thiomicrorhabdus aerobically and chemolithoautotrophically oxidizes sulfide to elemental sulfur.

It is known that in oxygen-free, sulfate-saturated layers beneath the sediment surface, sulfate-reducing microorganisms are among the most important participants that mediate a significant fraction of organic matter degradation (Yin et al., 2024). In the Bezymyanny cold spring, the highest distribution of sulfate-reducing bacteria in the bottom sediment, mainly belonging to phylum Thermodesulfobacteriota, was found. It should be noted that the large number of unclassified and uncultured representatives of sulfate-reducing bacteria suggests the presence of new species of sulfatereducing bacteria in the Bezymyanny cold spring.

## Conclusion

For the first time, the taxonomic diversity of microorganisms was studied, and characteristic features of microbial community structure of different biotopes (microbial mat, bottom sediment and water) in the cold sulfur spring Bezymyanny (Pribaikalsky district, Republic of Buryatia) were revealed by using molecular biological methods. According to the results of studies, sulfur-oxidizing (SOB) and sulfatereducing bacteria (SRB) were identified in the community, which indicates that sulfur cycling processes are taking place in the ecosystem of the Bezymyanny spring. On the whole, the analysis of taxonomic composition showed a high percentage of unclassified sequences in the communities studied. The obtained data indicate that the microbiota of cold sulfur springs is still a hidden resource of new taxa, including sulfur cycle bacteria. Studies of cold sulfur springs will further expand our knowledge of bacteria involved in the biogeochemical cycle of sulfur, their metabolism and evolution, and may indicate the ecological features of the development of sulfur microbial communities and their relationship to the environment of their habitat.

Data availability: all raw sequences obtained from 16S rRNA gene sequencing are available in the NCBI SRA archive under BioProject number PRJNA1202704

## Conflict of interest

The authors declare no conflict of interest.
